# TBC1D21 Potentially Interacts with and Regulates Rap1 during Murine Spermatogenesis

**DOI:** 10.3390/ijms19113292

**Published:** 2018-10-23

**Authors:** Chih-Chun Ke, Ying-Hung Lin, Ya-Yun Wang, Ying-Yu Wu, Mei-Feng Chen, Wei-Chi Ku, Han-Sun Chiang, Tsung-Hsuan Lai

**Affiliations:** 1 PhD Program in Nutrition & Food Science, Fu Jen Catholic University, New Taipei City 24205, Taiwan; koacurtis@gmail.com; 2 Department of Urology, En Chu Kong Hospital, New Taipei City 23702, Taiwan; 3 Graduate Institute of Biomedical and Pharmaceutical Science, Fu-Jen Catholic University, New Taipei City 24205, Taiwan; 084952@mail.fju.edu.tw (Y.-H.L.); l43219713@hotmail.com (Y.-Y.W.); 053824@mail.fju.edu.tw (H.-S.C.); 4 Department of Chemistry, Fu Jen Catholic University, New Taipei City 24205, Taiwan; vic0009@gmail.com; 5 Bone and Joint Research Center, Chang Gung Memorial Hospital, Taoyuan County 33305, Taiwan; mfchen0@gmail.com; 6 School of Medicine, Fu Jen Catholic University, New Taipei City 24205, Taiwan; 089052@mail.fju.edu.tw; 7 Department of Obstetrics and Gynecology, Cathay General Hospital, Taipei 10630, Taiwan; 8 Institute of Systems Biology and Bioinformatics, National Central University, Jhongli City, Taoyuan County 32001, Taiwan

**Keywords:** Rap1, TBC1D21, spermatids, male infertility

## Abstract

Few papers have focused on small guanosine triphosphate (GTP)-binding proteins and their regulation during spermatogenesis. *TBC1D21* genes (also known as male germ cell RAB GTPase-activating protein MGCRABGAP) are related to sterility, as determined through cDNA microarray testing of human testicular tissues exhibiting spermatogenic defects. TBC1D21 is a protein specifically expressed in the testes that exhibits specific localizations of elongating and elongated spermatids during mammalian spermiogenesis. Furthermore, through co-immunoprecipitation (co-IP) and nano liquid chromatography–tandem mass spectrometry (nano LC–MS/MS), Rap1 has been recognized as a potential TBC1D21 interactor. This study determined the possible roles of Rap1 and TBC1D21 during mammalian spermiogenesis. First, the binding ability between Rap1 and TBC1D21 was verified using co-IP. Second, the stronger signals of Rap1 expressed in elongating and elongated murine spermatids extracted from testicular sections, namely spermatogonia, spermatocytes, and round spermatids, were compared. Third, Rap1 and TBC1D21 exhibited similar localizations at postacrosomal regions of spermatids and at the midpieces of mature sperms, through isolated male germ cells. Fourth, the results of an activating Rap1 pull-down assay indicated that TBC1D21 overexpression inactivates Rap1 activity in cell models. In conclusion, TBC1D21 may interact with and potentially regulate Rap1 during murine spermatogenesis.

## 1. Introduction

### 1.1. Small Guanosine Triphosphate-Binding Proteins and GTPase-Activating Proteins

GTPase-activating proteins (GAPs) and guanine nucleotide exchange factors (GEFs) regulate the biological status of small guanosine triphosphate (GTP)-binding proteins [1,2]. GAPs activate the GTPase domains of small GTP-binding proteins to enable transitions from the GTP-bound active forms to the guanosine diphosphate (GDP)-bound inactive forms. For small GTP-binding proteins, GEFs stimulate GDP-for-GTP exchanges. The guanine nucleotide dissociation inhibitors bind to the small GTP-binding protein to prevent the nucleotide exchange of GDP-bound inactive forms. Furthermore, the group of small GTP-binding proteins contains more than 100 members, such as Ras, Rho, Ran, Rab, Rap, Ral, and Rhe [3]. Small GTP-binding proteins regulate almost all aspects of cellular functions, including proliferation, survival, apoptosis, cytoskeleton modulation, cell polarity, intracellular vesicular traffic and secretion, contact with neighboring cells, and contact with extracellular matrices. [3,4]. Dysregulated small GTP-binding and its regulators are involved in several diseases, such as cancer, endocrine diseases, and reproductive diseases [5,6].

### 1.2. Identification and Characterization of a Novel GTPase-Activating Protein: TBC1D21

We previously identified a novel sterility-related GAP gene through cDNA microarray, which compared testicular tissues from human fertile and Sertoli-cell-only syndrome cases [7]. The novel GAP contains a conserved RabGAP catalytic domain, Tre2/Bub2/Cdc16 (TBC). This GAP is specifically expressed in post-meiotic male germ cells. It localizes particularly at the peri-acrosomal and manchette regions of elongating and elongated spermatids and at the tail regions of mature spermatozoa [8]. Furthermore, its GTPase-activating activity was demonstrated using a GTPase-activating assay [9]. We identified Rab10, Rab5C, Rab1A, and Rap1 as its potential interactors and substrates through co-immunoprecipitation (co-IP) and nano liquid chromatography–tandem mass spectrometry (nano LC–MS/MS) [9]. On the basis of its specific expression in male germ cells during murine and human spermiogenesis, as well as its potential Rab-specific function, it also has been named previously as male germ cell Rab GTPase-Activating protein (MGCRABGAP) [8].

### 1.3. Rap1 Function in Male Reproduction

Rap1 is a small GTPase protein involved in controlling different cellular processes, including proliferation, differentiation, apoptosis prevention, and cell adhesion [10]. Rap1 has recently been implicated in the proper proliferation and differentiation of testicular germ cells and the release of mature sperm from Sertoli cells [11,12,13]. In mice, overexpression of inactive Rap1 mutant resulted in (1) decreased sperm number and motility, (2) underdeveloped male germ cells released prematurely from the supporting Sertoli cells, and (3) a high percentage of sperm with abnormal head morphology [14]. Abnormal sperm heads and spermiation seem to be caused by apical spermatids that destabilize Sertoli cells [15,16]. In a clinical study, Yang et al., reported that *Rap1* transcripts isolated from human azoospermic testes are significantly upregulated relative to controls [17]. These results suggest that *Rap1* genes are potentially associated with sterility-related genes.

In this study, we determined the possible interactions and regulation of TBC1D21 and Rap1 during murine spermiogenesis.

## 2. Results

### 2.1. TBC1D21 Interacts with Rap1 in NTERA-2 cl.D1 Cells

**Figure 1:**
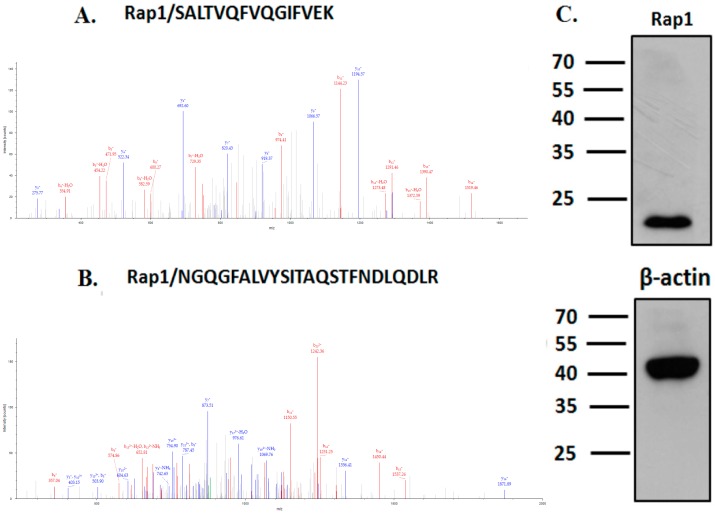
Identification of interactors of TBC1D21 through co-immunoprecipitation (co-IP) and nano LC–MS/MS. (**A**,**B**) Tandem mass spectrometry (MS/MS) spectrum of the tryptic peptides of Rap1: SALTVQFVQGIFVEK and NGQGFALVYSITAQSTFNDLQDLR. (**C**) Immunoblotting (IB) of NTERA-2 cl.D1 (NT2D1) cell lysates with the anti-Rap1 and anti-β-actin antibodies.

**Figure 2:**
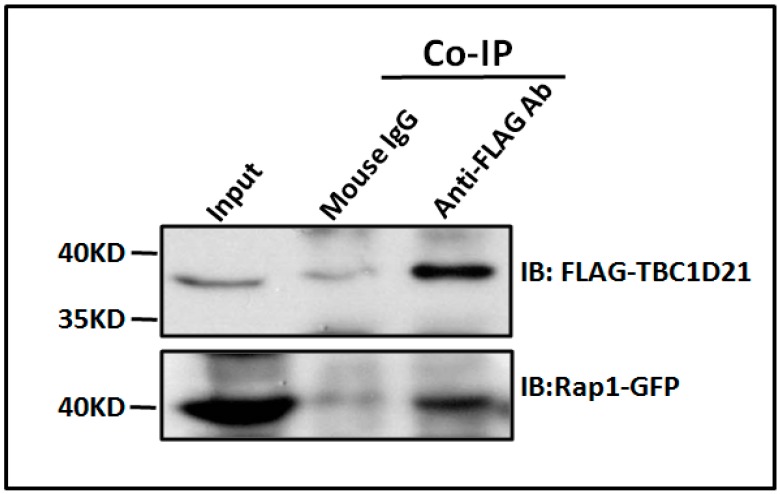
TBC1D21 interacts and with Rap1. The co-IP of FLAG-TBC1D21 with Rap1-GFP. The lysates of NTERA-2 cl.D1 (NT2D1) cells transfected with pEGFP-Rap1, and a pFLAG-TBC1D21 vector was subjected to immunoprecipitation with an anti-FLAG antibody (Lane 3) or a nonspecific control IgG (Lane 2), followed by IB with the anti-FLAG antibody (top panel) or anti-Green fluorescent protein (GFP)antibody (bottom panel). An input protein (5%; Lane 1) was used as a control during IB.

In our previous study, we identified several small G proteins as possible TBC1D21 interactors through co-immunoprecipitation (co-IP) and nano LC–MS/MS [9]. Rap1 is an interactor of TBC1D21. Two Rap1 peptides have been identified (Figure 1A,B). Moreover, Rap1 was strongly expressed in NTERA-2 cl.D1 (NT2D1) cells, a pluripotent human testicular embryonal carcinoma cell line (Figure 1C). To confirm that TBC1D21 interacts with Rap1, co-IP was performed. The co-IP and immunoblotting (IB) results in Figure 2 confirm that TBC1D21 interacts with Rap1: The lysates of cells co-transfected with the pFLAG-TBC1D21 and pEGFP-Rap1 plasmids were co-immunoprecipitated and immunoblotted using anti-FLAG (Figure 2, upper panel) and anti-GFP (Figure 2, bottom panel) antibodies.

### 2.2. Expression Patterns of Rap1 during Murine Spermatogenesis

**Figure 3:**
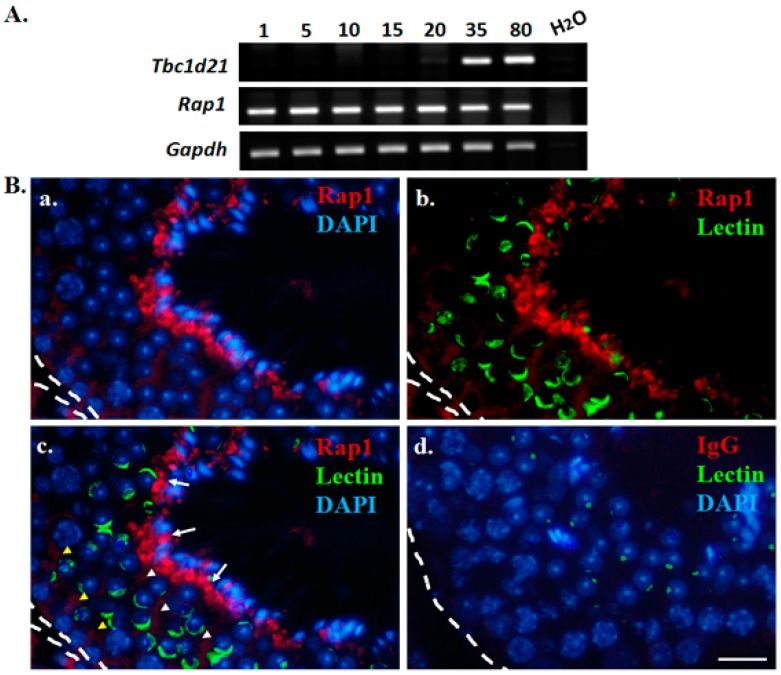
Expression patterns of *Rap1* during murine spermatogenesis. (**A**) Expression patterns of *Rap1* and *Tbc1d21* transcripts in the mouse testes of different postnatal days, determined by RT-PCR. *Gapdh* was the loading control. (**B**) Localization of Rap1 in testicular sections of an adult (80 days old) mouse, as determined through an immunofluorescence (IF) assay. Anti-Rap1 (**a**–**c**) or mouse IgG (**d**) antibodies (red); acrosomal marker (green; **b**,**c**); 4′,6-diamidino-2-phenylindole (DAPI) (blue; **a**, **c**, and **d**); merging of Rap1 and DAPI (**a**); merging Rap1 and acrosomal marker (**b**); merging of Rap1, acrosomal marker, and DAPI (**c**); merging of mouse IgG control, acrosomal marker, and DAPI (**d**). Scale bar: 20 μm. The discontinuous white line marks the edge of the seminiferous tubules within the murine testicular tissue. Yellow arrowheads: Rap1 signals in spermatogonia, spermatocytes, and round spermatids; White arrows: Rap1 signals around the heads of elongating spermatids.

To determine the expression patterns of *Rap1* transcripts during the different developmental stages, testicular tissues were retrieved from mice on different postnatal days. In the mouse testes, male germ cell populations within the seminiferous tubules develop into different stages on specific postnatal days (days 1–5: spermatogonia; day 10: primary spermatocytes; day 15: pachytene spermatocytes; day 20: round spermatids; day 35: elongating spermatids; days 80: mature spermatozoa) [18]. The reverse transcription polymerase chain reaction (RT-PCR) results (Figure 3A) indicated that *Tbc1d21* transcripts were expressed in murine testes from postnatal day 35. *Rap1* transcripts appear from day 1 to day 80 of the murine spermatogenesis process. Rap1 localization during murine spermatogenesis was determined through immunofluorescence (IF) assays on the testicular sections. We discovered that Rap1 was slightly expressed around the spermatogonia, spermatocytes, and round spermatids (Figure 3B, yellow arrowhead). The high-intensity signals for Rap1 were localized around the heads of elongating spermatids (Figure 3Bc; white arrows). The results indicated that Rap1 is involved in murine spermatogenesis.

### 2.3. Dynamic Localization of Rap1 and TBC1D21 during Murine Spermiogenesis

**Figure 4:**
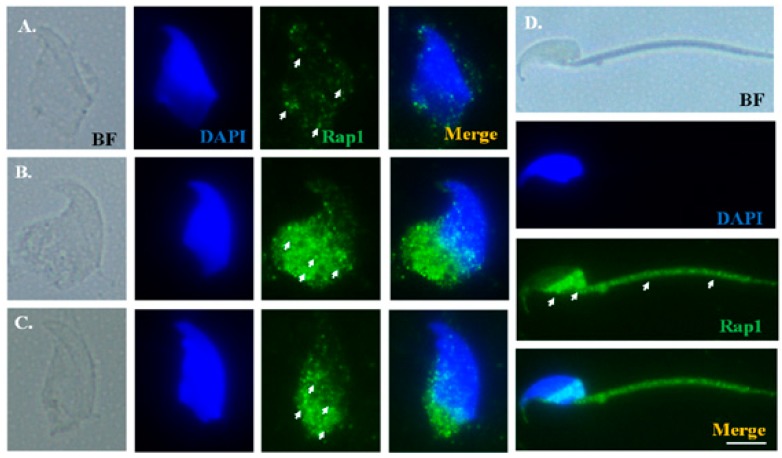
Rap1 signals showed multiple localizations during murine spermiogenesis. From left to right: bright field (BF), DAPI staining (blue), and Rap1 signal (green), as well as the merging of Rap1 and DAPI at early elongation (**A**), late elongation (**B**), early elongated spermatids (**C**), and mature sperm (**D**). Arrows indicate Rap1 signals. Scale bar: 5 μm.

**Figure 5:**
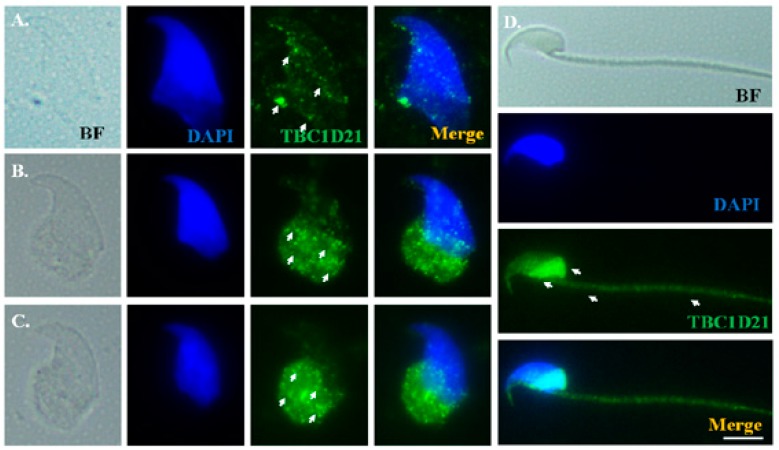
TBC1D21 signals showed multiple localizations during murine spermiogenesis. From left to right: Bright field (BF), DAPI staining (blue), and TBC1D21 signal (green), as well as the merging of TBC1D21 and DAPI at early elongation (**A**) and late elongation (**B**), early elongated spermatids (**C**), and mature sperm (**D**). Arrows indicate TBC1D21 signals. Scale bar: 5 μm.

To precisely elucidate Rap1 localization during murine spermiogenesis, testicular germ cell populations and mature spermatozoa were isolated and subjected to IF staining. In addition, dissected sperm heads were used to declare the different developmental stages of spermatids through 4′,6-diamidino-2-phenylindole (DAPI) staining. During the elongation phase, weak Rap1 signals were noted around sperm heads (Figure 4A) of the elongating spermatids, and concentrated around the postacrosomal regions of the elongated spermatids (Figure 4B,C). In mature spermatozoa, Rap1 signals were localized under the perinuclear region and midpiece (Figure 4D). TBC1D21 exhibited patterns of distribution highly similar to those of Rap1 (Figure 5).

### 2.4. Rap1 Is Regulated by TBC1D21

**Figure 6:**
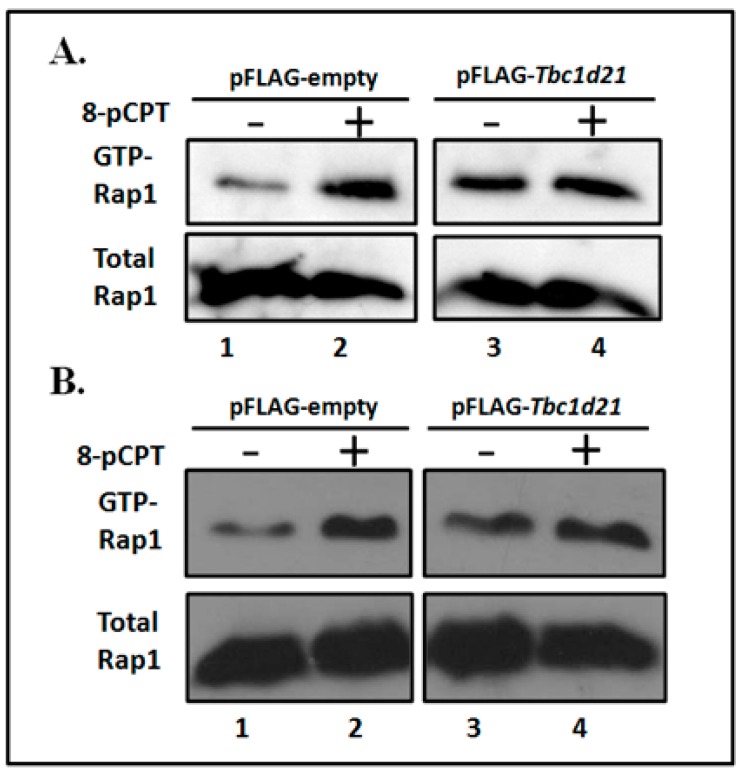
TBC1D21 regulates Rap1 activity. (**A**,**B**) NT2D1 cells were transfected with pFLAG-empty (Lanes 1 and 2) or pFLAG-*Tbc1d21* (Lanes 3 and 4). Cell lysates were subjected to Rap1 activation through treatment with 8-pCPT-2′-O-Me-cAMP (8-pCPT) (Lanes 2 and 4) or mock treatment (Lanes 1 and 3). Following IB with the anti-Rap1 antibody (upper panel), activated Rap1 was incubated with the Glutathione S-transferases (GST)-Rap-binding domain, and then pulled down using anti-GST antibodies. An equal amount of cell lysates were immunoblotted with the anti-Rap1 antibody as a control. “+”: add 8-pCPT; “−”: absent 8-pCPT.

To confirm the GTPase-activating effects of TBC1D21 on Rap1, the pull-down process for activating Rap1 was applied in NT2D1 cells. To induce Rap1 activation, a membrane-permeable GTP analog, 8-pCPT-2′-*O*-Me-cAMP (8-pCPT), was used in this assay, as reported previously [12,13,19]. In the experimental group, the 8-pCPT induction increased activated Rap1 levels (Figure 6A,B, Lane 2) compared with the controls (Figure 6A,B, Lane 1). In the overexpressed TBC1D21 group, Rap1 signals lost the inducing effects in Lane 4 relative to Lane 3 (Figure 6A,B). Figure 6 indicates that TBC1D21 may have inactivated Rap1 activity in the cell.

## 3. Discussion

In this study, we characterized Rap1 as a substrate of TBC1D21 through co-IP and an activated Rap1 pull-down assay. We also determined the spatiotemporal expression and localization of Rap1 through isolation of murine male germ cells during mammalian spermiogenesis. On the basis of these results, we suggest that TBC1D21 potentially interacts with and may regulate Rap1 in the testes, and this may play a role in spermatogenesis.

### 3.1. TBC1D21 Regulates Rabs and Other Small GTPases

In humans and mice, more than 42 proteins contain conserved RabGAP domains, the catalytic domains of Tre2/Bub2/Cdc16 (TBC) [2,20]. Over the past 10 years, accumulating evidence has indicated that RabGAPs act as dominant regulatory nodes, involved in integrating signaling between Rabs and other small GTPases [20,21]. For instance, USP6/TRE17 is a RabGAP that interacts with Arf6 GEFs to facilitate the movement of Arf6 to the plasma membrane [22]. Similarly, TBC1D3 also joins an Arf6-dependent pathway in macropinocytosis [23]. Furthermore, TBC1D2A is an effector of Rac1, and TBC1D10C exhibits Ras-GAP activity [24,25]. We previously identified TBC1D21 interactors and proved that one of them, Rab10, is involved in the formation of the manchette structure during sperm head formation [9]. In the present study, we determined that TBC1D21 may interact with and regulate Rap1.

### 3.2. Molecular and Cellular Functions of Rap1 during Mammalian Spermatogenesis

Rap1 was initially found in mouse spermatids; it forms complexes with B-Raf/14-3-3 φ complexes [11]. It was also proven that Rap1 is expressed at murine spermatocytes [13,26]. Aivatiadou et al. generated transgenic mice with a Rap1-inactivated mutation to discover possible Rap1 functions. The immature germ cells in the mutated mice were collapsed from Sertoli cells and exhibited heads with abnormal morphology [14]. Berruti claimed that Rap1 regulates spermiation in apical ectoplasmic cases; this was presumed to operate through Cdc42/actin, Cdc42/Par-JamC, or Afadin/Nectin-3 complexes [16]. However, whether or not Rap1 is involved in differentiating spermatids is still unknown. In this study, we found that Rap1 localizes the multiplex signals during the formation of sperm heads and sperm tails (Figure 3). It provides another possible explanation of the causes of abnormal sperm heads in Rap1-mutated mice.

### 3.3. GTPase-Activating Proteins and Other Small GTPases during Mammalian Spermiogenesis

The enzymatic functions of Rap1 are regulated by GEFs and GAPs. Several GEFs (e.g., EPAC, C3G, PDZ-GEFs, CalDAGs, and RapGEF6) have been indicated as activating Rap1 for GTP forms in different cells [12,27,28,29,30,31]. Two of them, EPAC and RapGEF6, are involved in male germ cells [13,30]. EPAC (exchange protein directly activated by cAMP) is colocalized with Rap1 within murine spermatocytes and round spermatids. It also promotes Rap1 activation in cultured cells [13]. Knockout of another GEF, RapGEF6, results in male infertility, and accounts for reduced testis size as well as reduced sperm count and motility. Moreover, the knockout also produces high percentages of morphologically abnormal sperm [30]. Several GAPs are involved in the inactivation of Rap1 in non-germ cell lines (e.g., Rap1-GAP1, Rap1-GAP2, Spa1, and SPAR1,2,3) [27]. However, investigational evidence for Rap1-GAPs in the testis has not been reported thus far; for the first time, we propose that TBC1D21 potentially regulates Rap1.

### 3.4. TBC1D21 and Rap1 in Male Infertility

Infertility affects approximately 10% of couples globally; of these, approximately 30% are infertile because of male infertility [32,33]. Male infertility is a complex condition, including anatomical pathologies, environmental exposure, and genetic deficiency. However, the genetic and molecular pathological mechanisms are generally unknown [32]. A clinical study, including hypospermatogenesis, maturation arrest, and Sertoli-cell-only cases, reports that *TBC1D21* transcript levels in testicular tissue are lower in infertile men than in fertile men [8]. However, *Rap1A* mRNA expression is higher in the testicular tissues of men with hypospermatogenesis and maturation arrest than in that of fertile men [17]. Thus, infertile men have decreased *TBC1D21* expression but increased *Rap1A* expression—a negative correlation. This may indicate the loss of *TBC1D21* expression after increase in *Rap1* levels. However, further research on this is warranted.

## 4. Materials and Methods

### 4.1. Liquid Chromatography–Tandem Mass Spectrometry, Co-Immunoprecipitation, and Immunoblotting

Identification of TBC1D21 interactors was performed through co-IP and following LC–MS/MS, as described previously [9,10]. To test whether TBC1D21 was actually binding with Rap1, co-IP analysis and IB were performed. NT2D1 cells were co-transfected with pFLAG-*TBC1D21* and pEGFP-*RAP1* plasmids by using Lipofectamine 3000 reagent (Cat No. 1816988; Invitogene, Waltham, MA, USA). Cell lysate (4 mg in 1 mL) was prepared and mixed with protein A/G beads (Santa Cruz Biotechnology Inc., Santa Cruz, CA, USA) for 1 h at 4 °C on a rotator (15 rpm). Next, the clear supernatant was isolated through centrifugation at 1000× *g* for 30 s at 4 °C; it was mixed overnight with control immunoglobulin G and an anti-FLAG antibody (Cat No. F1804; Sigma-Aldrich, St. Louis, MO, USA) at 4 °C on a rotator (15 rpm). The immunoprecipitated samples were collected through centrifugation (1000× *g* for 1 h at 4 °C) and washed twice with phosphate-buffered saline. Subsequently, IB was performed using anti-FLAG (Cat No. F1804; Sigma-Aldrich, St. Louis, MO, USA) and anti-EGFP antibodies (Cat No. ab290; Abcam, Cambridge, MA, USA). This was followed by immunoblotting with a secondary antibody conjugated with horseradish peroxidase (HRP), after which samples were exposed on an X-ray film.

### 4.2. Real-Time Polymerase Chain Reaction

Total RNA was extracted from mice testes on postnatal day 1, day 5, day 10, day 15, day 20, day 35, and day 80 and above (adult mice). The conditions for real-time polymerase chain reaction (RT-PCR) and how product detection was performed were described in our previous publication [8]. The primer pairs used were MgcRabGAP (F′: ctgcctgcgtggtttattct; R′: gcaggtgtccattgtgagtg) and Rap1 (F′: ccagtggaaaagaagaagccta; R′: gaatgctggcactttccaat). 

### 4.3. Rap1 Pull-Down Assay

NT2D1 cells were transfected with pFLAG-empty or pFLAG-*TBC1D21* plasmids by using transfection reagents. After 48 h, the cell lysates were incubated with 200 μM 8-pCPT for 10 min to activate Rap1. The mixtures were incubated with a GST–Rap-binding domain and then pulled down to activate Rap1 through anti-GST antibodies; the mixtures were subsequently activated for IB with anti-Rap1 antibodies. All steps were performed according to the manufacturer protocols. The experiment used an Active Rap1 Pull-Down and Detection Kit (Cat No.: 16120; Sigma-Aldrich, St. Louis, MO, USA). Equal amounts of cell lysates were analyzed through IB with an anti-Rap1 antibody to normalize the total Rap1 content.

### 4.4. Isolated Testicular Germ Cells and Immunofluorescence Assay

The mouse studies were approved by the Institutional Animal Care and Use Committee of Fu Jen Catholic University (A10180, 1 May 2013). Testes of adult C57BL/6 male mice were isolated. Male germ cells were separated using various centrifugal forces, based on the densities of different types of germ cells; the protocol was modified from a previous protocol [34,35]. After decapsulation of murine testis, the seminiferous tubules were broken down in a DMEM/F12 medium. The mixture was augmented with 1 mg/mL trypsin, 0.75 mg/mL collagenase, 5 μg/mL DNAase I, 1× protease inhibitor cocktail (Sigma-Aldrich, Shanghai, China), and 1× antibiotics (Invitrogen, Waltham, MA, USA), and was then incubated for 1.5 h at 37 °C under shaking at 140 rpm. To exclude any undigested tissue, these mixtures were filtered through 35-μM nylon filters. Four types of the suspension solutions were collected after centrifugation at 700, 400, 200, and 100× *g*. Finally, the cell pellets were collected from these suspensions after centrifugation at 3000× *g*; the cell pellets were spread on a slide. Mature spermatozoa were collected from the vas deferens of adult male mice. The current IF assay protocols were identical to those of our previous studies [8]. Both anti-MGCRABGAP (Cat No. ab119060; Abcam, Cambridge, MA, USA), and anti-RAP1 (Cat No.: ab175329; Abcam, Cambridge, MA, USA) were used for double staining. Lectin peanut agglutinin conjugated to Alexa Fluor 568 (Invitrogen, Waltham, MA, USA) was used to locate the acrosomes of the male germ cells, whereas DAPI was used to stain the nuclei.

## 5. Conclusions

For the first time, we suggest that TBC1D21 may interact with and regulate Rap1, and that Rap1 may be involved in mouse spermatogenesis.

## References

[1] Bos J.L., Rehmann H., Wittinghofer A. (2007). GEFs and GAPs: Critical elements in the control of small G proteins. Cell.

[2] Pan X., Eathiraj S., Munson M., Lambright D.G. (2006). TBC-domain GAPs for Rab GTPases accelerate GTP hydrolysis by a dual-finger mechanism. Nature.

[3] Mollinedo F., Perez-Sala D., Gajate C., Jimenez B., Rodriguez P., Lacal J.C. (1993). Localization of rap1 and rap2 proteins in the gelatinase-containing granules of human neutrophils. FEBS Lett..

[4] Lucking R., Lawrey J.D., Gillevet P.M., Sikaroodi M., Dal-Forno M., Berger S.A. (2014). Multiple ITS haplotypes in the genome of the lichenized basidiomycete Cora inversa (Hygrophoraceae): Fact or artifact?. J. Mol. Evol..

[5] Mukhopadhyay A., Nieves E., Che F.Y., Wang J., Jin L., Murray J.W., Gordon K., Angeletti R.H., Wolkoff A.W. (2011). Proteomic analysis of endocytic vesicles: Rab1a regulates motility of early endocytic vesicles. J. Cell Sci..

[6] Liu Z.L., Wu H., Tang B.H., Qiu S., Li Z.L. (2013). Atmospheric corrections of passive microwave data for estimating land surface temperature. Opt. Express.

[7] Lin Y.H., Lin Y.M., Teng Y.N., Hsieh T.Y., Lin Y.S., Kuo P.L. (2006). Identification of ten novel genes involved in human spermatogenesis by microarray analysis of testicular tissue. Fertil. Steril..

[8] Lin Y.H., Lin Y.M., Kuo Y.C., Wang Y.Y., Kuo P.L. (2011). Identification and characterization of a novel Rab GTPase-activating protein in spermatids. Int. J. Androl..

[9] Boersema P.J., Raijmakers R., Lemeer S., Mohammed S., Heck A.J. (2009). Multiplex peptide stable isotope dimethyl labeling for quantitative proteomics. Nat. Protoc..

[10] Chen K.C., Lin C.M., Huang C.J., Chen S.K., Wu S.T., Chiang H.S., Ku W.C. (2016). Dual Roles of 17-beta Estradiol in Estrogen Receptor-dependent Growth Inhibition in Renal Cell Carcinoma. Cancer Genom. Proteom..

[11] Mori-Quiroz L.M., Shimkin K.W., Rezazadeh S., Kozlowski R.A., Watson D.A. (2016). Copper-Catalyzed Amidation of Primary and Secondary Alkyl Boronic Esters. Chemistry.

[12] Zhang L., Zhang H., Liu M., Dong B. (2016). Reprogrammable Logic Gate and Logic Circuit Based on Multistimuli-Responsive Raspberry-like Micromotors. ACS Appl. Mater. Interfaces.

[13] Pizzochero M., Bonfanti M., Martinazzo R. (2016). Hydrogen on silicene: Like or unlike graphene?. Phys. Chem. Chem. Phys..

[14] Aivatiadou E., Mattei E., Ceriani M., Tilia L., Berruti G. (2007). Impaired fertility and spermiogenetic disorders with loss of cell adhesion in male mice expressing an interfering Rap1 mutant. Mol. Biol. Cell.

[15] Chen Y.T., Holcomb C., Moore H.P. (1993). Expression and localization of two low molecular weight GTP-binding proteins, Rab8 and Rab10, by epitope tag. Proc. Natl. Acad. Sci. USA.

[16] Tan Y.S., Kim M., Kingsbury T.J., Civin C.I., Cheng W.C. (2014). Regulation of RAB5C is important for the growth inhibitory effects of MiR-509 in human precursor-B acute lymphoblastic leukemia. PLoS ONE.

[17] Kitayama H., Sugimoto Y., Matsuzaki T., Ikawa Y., Noda M. (1989). A ras-related gene with transformation suppressor activity. Cell.

[18] Yang Z.W., Wreford N.G., de Kretser D.M. (1990). A quantitative study of spermatogenesis in the developing rat testis. Biol. Reprod..

[19] Rappsilber J., Mann M., Ishihama Y. (2007). Protocol for micro-purification, enrichment, pre-fractionation and storage of peptides for proteomics using StageTips. Nat. Protoc..

[20] Mukhopadhyay A., Quiroz J.A., Wolkoff A.W. (2014). Rab1a regulates sorting of early endocytic vesicles. Am. J. Physiol. Gastrointest. Liver Physiol..

[21] Frasa M.A., Koessmeier K.T., Ahmadian M.R., Braga V.M. (2012). Illuminating the functional and structural repertoire of human TBC/RABGAPs. Nat. Rev. Mol. Cell Biol..

[22] Cornwall G.A. (2009). New insights into epididymal biology and function. Hum. Reprod. Update.

[23] Duan R.B., Zhang L., Chen D.F., Yang F., Yang J.S., Yang W.J. (2014). Two p90 ribosomal S6 kinase isoforms are involved in the regulation of mitotic and meiotic arrest in Artemia. J. Biol. Chem..

[24] Wolgemuth D.J., Laurion E., Lele K.M. (2002). Regulation of the mitotic and meiotic cell cycles in the male germ line. Recent Prog. Horm. Res..

[25] Grallert B., Sipiczki M. (1991). Common genes and pathways in the regulation of the mitotic and meiotic cell cycles of Schizosaccharomyces pombe. Curr. Genet..

[26] Larance M., Ramm G., Stockli J., van Dam E.M., Winata S., Wasinger V., Simpson F., Graham M., Junutula J.R., Guilhaus M. (2005). Characterization of the role of the Rab GTPase-activating protein AS160 in insulin-regulated GLUT4 trafficking. J. Biol. Chem..

[27] Han Y., Qin G., Jungemann C., Hu M. (2016). Strain-modulated electronic and thermal transport properties of two-dimensional O-silica. Nanotechnology.

[28] Kwon W.S., Rahman M.S., Ryu D.Y., Park Y.J., Pang M.G. (2015). Increased male fertility using fertility-related biomarkers. Sci. Rep..

[29] Decker C.E., Yang Z., Rimer R., Park-Min K.H., Macaubas C., Mellins E.D., Novack D.V., Faccio R. (2015). Tmem178 acts in a novel negative feedback loop targeting NFATc1 to regulate bone mass. Proc. Natl. Acad. Sci. USA.

[30] Martinolich A.J., Neilson J.R. (2014). Pyrite formation via kinetic intermediates through low-temperature solid-state metathesis. J. Am. Chem. Soc..

[31] Cendoya E., Farnochi M.C., Chulze S.N., Ramirez M.L. (2014). Two-dimensional environmental profiles of growth and fumonisin production by Fusarium proliferatum on a wheat-based substrate. Int. J. Food Microbiol..

[32] O’Flynn O’Brien K.L., Varghese A.C., Agarwal A. (2010). The genetic causes of male factor infertility: A review. Fertil. Steril..

[33] Ferlin A., Raicu F., Gatta V., Zuccarello D., Palka G., Foresta C. (2007). Male infertility: Role of genetic background. Reprod. Biomed. Online.

[34] Lin Y.H., Lin Y.M., Wang Y.Y., Yu I.S., Lin Y.W., Wang Y.H., Wu C.M., Pan H.A., Chao S.C., Yen P.H. (2009). The expression level of septin12 is critical for spermiogenesis. Am. J. Pathol..

[35] Yeh Y.C., Yang V.C., Huang S.C., Lo N.W. (2005). Stage-dependent expression of extra-embryonic tissue-spermatogenesis-homeobox gene 1 (ESX1) protein, a candidate marker for X chromosome-bearing sperm. Reprod. Fertil. Dev..

